# Connexin‐50 role in spreading of polymorphic alpha‐synuclein in Synucleiopathy

**DOI:** 10.1002/alz70855_105939

**Published:** 2025-12-24

**Authors:** Nicha Puangmalai, Urmi Sengupta, Nemil Bhatt, Abbigael Aday, Fadhl Al‐Shaebi, Cynthia Jerez, Nikita Shchankin, Jai Yi Liew, Mauro Montalbano, Rakez Kayed

**Affiliations:** ^1^ Mitchell Center for Neurodegenerative Diseases, University of Texas Medical Branch, Galveston, TX, USA; ^2^ University of Texas Medical Branch, Galveston, TX, USA

## Abstract

**Background:**

Connexins, gap junction components, have been implicated in intercellular connectivity under physiological and pathophysiological conditions, including neurodegeneration. Synucleinopathies comprise a diverse group of neurodegenerative disorders pathologically characterized by α‐synuclein (α‐Syn) aggregates. However, little is known about connexin‐associated α‐Syn pathological spread in synucleinopathies

**Method:**

Here, we present evidence of connexin‐50 (Cx50) directly interacting with α‐Syn aggregates in human brains affected by synucleinopathies, including Alzheimer's disease (AD), dementia with Lewy bodies (DLB), and Parkinson's disease (PD). We also observed this interaction in pre‐clinical α‐Syn mouse models that exhibit Parkinson's disease‐related phenotypes. To achieve this, we utilized immunohistochemistry, pharmacological, and genetic manipulation techniques in connexin cell models and primary co‐cultures.

**Result:**

Utilizing well‐characterized α‐Syn oligomers (BDSOs) isolated from brains affected by Alzheimer's disease (AD), Lewy body dementia (DLB), and Parkinson's disease (PD), we demonstrate that BDSOs preferentially enter cells expressing connexin 50 (Cx50), a protein found in gap junctions, as confirmed by pharmacological inhibition. In live‐cell imaging experiments, we observed a significant reduction in BDSO uptake in primary neurons‐astrocytes co‐cultured from human wild‐type α‐Syn transgenic mice expressing genetically modified Cx50. This reduction was accompanied by a decrease in α‐Syn aggregates. Moreover, the downregulation of Cx50 was associated with a reduction in the activity of astrocytes, as evidenced by a decrease in pro‐inflammatory and an increase in anti‐inflammatory cytokines. These findings suggest that Cx50 plays a crucial role in the interplay between neurons and astrocytes.

**Conclusion:**

This study presents compelling evidence of the connection between disease‐relevant α‐Syn aggregates and Cx50 in neurons that regulate astrocyte activity. This insight sheds light on the intricate process of pathogenic α‐Syn spread in Synucleiopathies.

